# AI-Based Retinal Image Analysis for the Detection of Choroidal Neovascular Age-Related Macular Degeneration (AMD) and Its Association with Brain Health

**DOI:** 10.3390/brainsci15111249

**Published:** 2025-11-20

**Authors:** Chuying Shi, Jack Lee, Di Shi, Gechun Wang, Fei Yuan, Timothy Y. Y. Lai, Jingwen Liu, Yijie Lu, Dongcheng Liu, Bo Qin, Benny Chung-Ying Zee

**Affiliations:** 1 Centre for Clinical Research and Biostatistics, Jockey Club School of Public Health and Primary Care, Faculty of Medicine, The Chinese University of Hong Kong, New Territories, Hong Kong; 1155117139@link.cuhk.edu.hk (C.S.); jack@cuhk.edu.hk (J.L.); 2 Department of Ophthalmology, Zhongshan Hospital, Fudan University, Shanghai 200433, China; 14301050099@fudan.edu.cn (G.W.); yuan.fei@zs-hospital.sh.cn (F.Y.); 3 Department of Pathology, Fudan University Shanghai Cancer Centre, Shanghai 200433, China; 19111230021@fudan.edu.cn; 4 Hong Kong Eye Hospital, Department of Ophthalmology and Visual Sciences, The Chinese University of Hong Kong, New Territories, Hong Kong; tyylai@cuhk.edu.hk; 5 Shenzhen Aier Eye Hospital, Jinan University, Shenzhen 518020, China; liujingwen2@aierchina.com (J.L.); luyijie@aierchina.com (Y.L.); liudongcheng@aierchina.com (D.L.); qinbo@aierchina.com (B.Q.); 6 Shenzhen Aier Ophthalmic Technology Institute, Shenzhen 518057, China; 7 Aier School of Ophthalmology, Central South University, Changsha 410083, China

**Keywords:** age-related macular degeneration, artificial intelligence, colour fundus retinal image analysis, image segmentation

## Abstract

**Purpose**: This study aims to develop a method for detecting referable (intermediate and advanced) age-related macular degeneration (AMD) and neovascular AMD, as well as providing an automatic segmentation of choroidal neovascularisation (CNV) on colour fundus retinal images. We also demonstrated that brain health risk scores estimated by AI-based Retinal Image Analysis (ARIA), such as white matter hyperintensities and depression, are significantly associated with AMD and neovascular AMD. **Methods**: A primary dataset of 1480 retinal images was collected from Zhongshan Hospital of Fudan University for training and 10-fold cross-validation. Additionally, two validation subdataset comprising 238 images (retinal images and wide-field images) were used. Using fluorescein angiography-based labels, we applied the InceptionResNetV2 deep network with the ARIA method to detect AMD, and a transfer ResNet50_Unet was used to segment CNV. The risks of cerebral white matter hyperintensities and depression were estimated using an AI-based Retinal Image Analysis approach. **Results**: In a 10-fold cross-validation, we achieved sensitivities of 97.4% and 98.1%, specificities of 96.8% and 96.1%, and accuracies of 97.0% and 96.4% in detecting referable AMD and neovascular AMD, respectively. In the external validation, we achieved accuracies of 92.9% and 93.7% and AUCs of 0.967 and 0.967, respectively. The performances on two validation sub-datasets show no statistically significant difference in detecting referable AMD (*p* = 0.704) and neovascular AMD (*p* = 0.213). In the segmentation of CNV, we achieved a global accuracy of 93.03%, a mean accuracy of 91.83%, a mean intersection over union (IoU) of 68.7%, a weighted IoU of 89.63%, and a mean boundary F1 (BF) of 67.77%. **Conclusions**: The proposed method shows promising results as a highly efficient and cost-effective screening tool for detecting neovascular and referable AMD on both retinal and wide-field images, and providing critical insights into CNV. Its implementation could be particularly valuable in resource-limited settings, enabling timely referrals, enhancing patient care, and supporting decision-making across AMD classifications. In addition, we demonstrated that AMD and neovascular AMD are significantly associated with increased risks of WMH and depression.

## 1. Introduction

Worldwide, age-related macular degeneration (AMD) is the third most common cause of irreversible vision loss [1]. The global prevalence of AMD is projected to reach 288 million by 2040. This escalating burden underscores a significant public health issue with profound socioeconomic implications worldwide [2]. In addition, studies have shown that individuals with AMD are more likely to experience cognitive decline, dementia, and depression compared to those without AMD [3,4].

Clinically, AMD is classified as early-stage and late-stage [1,5]. The Age-Related Eye Disease Study (AREDS) demonstrated that, for patients with early-stage AMD and drusen, preventive strategies such as antioxidant vitamin supplements can reduce the risk of progression to late-stage AMD [6]. Therefore, identifying individuals with early-stage AMD for early intervention is crucial. Additionally, artificial intelligence (AI) approaches might relieve the medical burden and support future eye care services [7]. Recently, several studies have utilised artificial intelligence to detect AMD or its various stages using colour fundus retinal images, achieving good performance [8,9,10,11]. However, few of them can detect neovascular AMD and choroidal neovascularization (CNV), a key characteristic of neovascular AMD.

The neovascular AMD, involving choroidal neovascularization through Bruch’s membrane, leads to rapid central vision loss (over days or weeks), while the atrophic form advances slowly (over years or decades). It is this aggressive neovascular subtype that accounts for nearly 90% of all severe central vision loss, frequently causing permanent disability and legal blindness [12,13]. Consequently, it has a devastating effect on patients’ quality of life and functional independence [14]. A study found that when symptoms appeared less than 2 weeks earlier, 83% of lesions were potentially treatable [15]. If unrecognised and consequently untreated, CNV can rapidly deteriorate vision, and delays in intervention were significantly correlated with poorer short-term prognosis [16]. The National Institute for Health and Care Excellence (NICE) guideline in 2018 [17] recommended that people with neovascular AMD be urgently referred within one working day for a timely definitive diagnosis and treatment [18]. Therefore, detecting patients with neovascular AMD can help avoid rapid deterioration and improve vision.

The CNVs are also essential for AMD assessment and subsequent treatment [18]. The size of CNV at baseline is associated with visual acuity and contrast sensitivity and has well-recognised prognostic value [19,20,21,22]. Fluorescein angiography (FA) has been the gold standard for diagnosing AMD and confirming the presence of CNV [23]. FA can determine the extent, type, size, and location of CNV [13,23], and neither OCT (Optical Coherence Tomography) alone nor non-invasive OCT angiography (OCTA) can completely replace it [24]. However, potential risks of FA include tissue infiltration (if the drug extravasates into the vein) and allergic reactions, leading to adverse reactions such as nausea, vomiting, flushing, itching, hives, dyspnea, and syncope [25,26]. In addition to being time-consuming, they are less suitable than colour fundus retinal images for screening.

Colour fundus retinal photography is a quick, cost-effective screening tool. Therefore, we aim to train and validate our algorithms to detect referable AMD and neovascular AMD on colour fundus retinal images. The reference standard based on FA was used for the accurate ground truth. We also incorporated wide-angle retinal images and non-mydriatic images from different cameras for external validation. This approach can further confirm the generalizability of our algorithms across different imaging devices, showing their robustness and potential applicability in diverse clinical settings.

Furthermore, our algorithms were trained to automatically segment CNV on fundus retinal images, providing an explainable AI-based retinal image analysis (ARIA) result. Mapping the segmented CNV from fundus angiography onto fundus retinal images for algorithm training can combine the accuracy of fundus angiography with the cost-effectiveness of fundus retinal images as a screening tool. This ARIA approach for estimating visual parameters using segmented CNV offers potential insights that could support decision-making across AMD classifications, enhancing clinical utility in diverse practice environments.

Previous studies have suggested that AMD patients are at an increased risk for dementia and Alzheimer’s disease. However, the mechanism is still unclear. It has been suggested that reduced visual input due to macula impairment may impact brain structure and function. Others suggested that inflammatory pathways between AMD and dementia may contribute to the link. Studies also suggested that AMD increases the risk of depression due to vision loss, lifestyle changes, and social isolation. Since ARIA is capable of estimating WMH as a significant risk factor for dementia and Alzheimer’s diseases, and also the risk of depression based on the same retinal images for patients with AMD, it sheds light on the relationship between AMD and the risks of dementia and depression. These findings may suggest additional value to the AMD screening.

## 2. Method

### 2.1. Dataset

This study was approved by the Ethics Committee of Zhongshan Hospital of Fudan University (B2021-400R, 15 July 2021), and all methods adhered to the principles outlined in the Declaration of Helsinki.

#### 2.1.1. Primary Dataset

Patients who had been diagnosed with neovascular or non-neovascular AMD and taken the colour fundus retinal images between September 2018 and October 2021 at the Department of Ophthalmology, Zhongshan Hospital, Fudan University, were retrospectively collected. Digital 50° fundus images were captured using a mydriatic retinal camera (Topcon TRC-50DX, Tokyo, Japan), which enables multimodal fundus retinal imaging, including colour fundus retinal images, fundus autofluorescence (FAF), and fundus angiography (FA). All patients with neovascular AMD underwent examinations, including colour fundus retinal images, SD-OCT, FAF, and FA. To select a control, patients with or without a diagnosis of other eye diseases, such as diabetic retinopathy, glaucoma, or epiretinal membrane, were included. Images with poor image quality, caused by artefacts or other eye diseases such as cataracts and asteroid hyalosis, were excluded.

#### 2.1.2. Validation Dataset

The images used for the validation dataset were retrospectively collected from two separate databases. The first dataset is obtained from the patients at the Department of Ophthalmology, Zhongshan Hospital, Fudan University, from November 2021 to December 2023. A mydriatic retinal camera was used to take colour fundus retinal images with a 50° field of view (Topcon TRC-50DX, Tokyo, Japan), and non-mydriatic colour fundus retinal images with a 45° field of view (Topcon TRC-NW100, Tokyo, Japan). Another dataset was obtained from the patients’ database at the Shenzhen Aier Eye Hospital clinic from March 2022 to January 2023. A wide-field retinal camera took colour fundus retinal images with a 90° or 135° field of view (CLARUS 500, Carl Zeiss Meditec Inc., Dublin, CA, USA). The data collection and exclusion criteria were the same as those of the primary dataset. There is no overlap between the primary dataset and this validation dataset.

### 2.2. AMD Classification

There are four stages of the clinical classification: 1. no AMD; 2. early AMD; 3. intermediate AMD; and 4. advanced AMD [5]. Referable AMD was defined as intermediate and advanced AMD (Table 1). All images, with or without referable AMD, from the primary and validation datasets at Zhongshan Hospital were labelled by two ophthalmologists. Two other ophthalmologists labelled the images from Shenzhen Aier Eye Hospital. Among images with referable AMD, the labels of neovascular AMD or non-neovascular AMD were determined by clinical diagnosis. Neovascular AMD is characterised by choroidal neovascularization (CNV), which presents with features including fluid or retinal haemorrhage in various retinal layers, such as intraretinal or subretinal, or below the retinal pigment epithelium; retinal pigment epithelium detachments; hard exudates; or subretinal fibrous scar tissue [1]. Only the images with the same labels among ophthalmologists were included in the datasets.

CNVs can be identified by their fluorescence patterns [27] and segmented by drawing a manual contour that delineates the dye leakage associated with the CNV [28]. For CNV segmentation, two ophthalmologists manually segmented CNV by mapping retinal images onto FA in ImageJ (version 1.53t; National Institute of Health, USA). Two additional ophthalmologists, each with over 20 years of experience, reviewed them and then selected one at random. Only the images chosen by both ophthalmologists were included. If any reviewer disagreed with the segmentation, it would be discussed and revised.

### 2.3. Image Pre-Processing

To better visualise the lesions and detect AMD on images, image pre-processing was performed using three steps: (1) applying a method based on adapthisteq (https://www.mathworks.com/help/images/ref/adapthisteq.html, accessed on 17 February 2023) which can enhance the contrast of images by transforming the values using contrast-limited adaptive histogram equalisation (CLAHE); (2) cropping the images to a square shape; and (3) scaling the square images to 256 × 256 pixels.

### 2.4. Development of the Automatic Retinal Analysis Method for AMD

**Figure 1:**
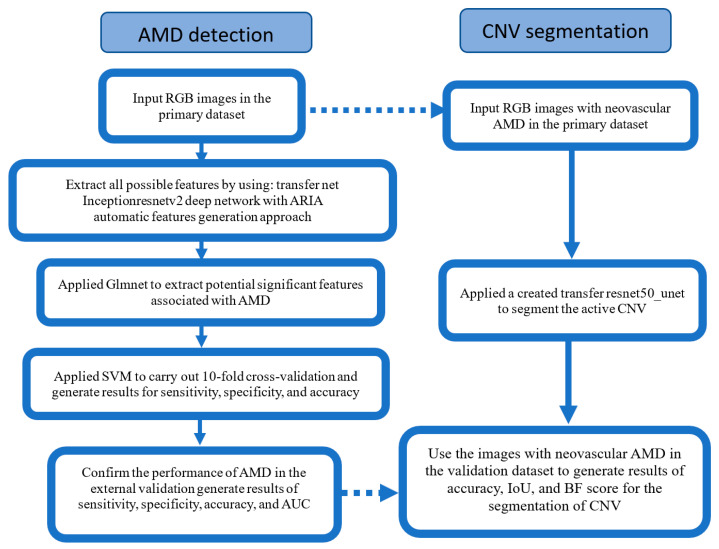
Flowchart of the presented method in AMD detection and CNV segmentation. Dotted arrow: only the images with neovascular AMD in the datasets were used for segmentation. AMD: Age-related macular degeneration; RGB: Red Green Blue colour code; ARIA: AI-based retinal image analysis; CNV: Choroidal neovascularisation; Glmnet: Generalised linear model via penalised maximum likelihood; SVM: Support vector machine; AUC: area under the curve; IoU: Intersection over union; BF: Boundary F1.

Retinal images were processed and analysed using the automatic retinal image analysis (ARIA) method, implemented in MATLAB R2023b [29]. To identify feature-relevant pixels, we employed Haralick texture feature analysis, fractal analysis, and a suite of modified pre-trained deep networks, including a modified transfer network (ResNet50). Our analysis (Figure 1) commenced with AMD detection using a transfer net, an InceptionResNetV2 deep network, where retinal images served as input, and the ‘predictions softmax’ layer generated the feature output. We then utilised the ARIA automatic feature-generation approach [29] to generate all possible features associated with AMD from pixel-level data. Subsequently, feature selection was performed using a generalised linear model with penalised maximum likelihood (Glmnet) to abstract the most AMD-relevant feature subsets. To enhance robustness and avoid overfitting, a 10-fold cross-validation support vector machine (SVM) was used. The final SVM model’s performance was evaluated on an external validation dataset.

For CNV segmentation, we utilised a U-Net architecture [30] and applied a pre-trained transfer ResNet50_UNet [31] to images from the primary dataset with neovascular AMD. The U-Net architecture is built from symmetric encoder and decoder sub-networks, comprising multiple stages (depth) and layers, connected by a bridge. After training, we used images of neovascular AMD from the validation dataset to evaluate performance.

### 2.5. Statistical Analysis

Machine learning and deep learning techniques were applied. The TransferNet InceptionResNetV2 convolutional neural network was utilised in MATLAB, with retinal images (RGB, size 299 × 299 × 3) serving as input. The generated features were extracted from the ‘predictions_softmax’ layer, based on pixels associated with AMD status [31]. Furthermore, texture, fractal, and spectrum-related features related to AMD were obtained by applying the Automatic Retinal Image Analysis (ARIA) algorithm developed in MATLAB. These features included high-order spectra and fractal dimensions [29]. Subsequently, the glmnet approach, implemented in MATLAB, was utilised to select a subset of features with the most significant relevance based on penalised maximum likelihood [32,33]. These refined features are strongly associated with AMD. To validate our feature selection models, a 10-fold cross-validation approach was used with the support vector machine (SVM) algorithm. The testing datasets used during cross-validation were distinct from those used for model training, thereby minimising the risk of overfitting [33,34]. At this stage, we calculated sensitivity, specificity, and accuracy using 10-fold cross-validation to evaluate classification performance in the primary dataset. Finally, we confirmed the performance of our model on an external dataset by calculating the area under the curve (AUC) of the receiver operating characteristic (ROC) curve. The method of Hanley and McNeil was used to compare the areas under two ROC curves [35]. For CNV segmentation, we also used mean accuracy, mean intersection over union (IoU), global accuracy, weighted IoU, and mean boundary F1 (BF) score to assess the performance of the automatic segmentation [36]. All analyses were performed using SPSS 20 and MATLAB 2020a software.

To estimate the early risk of dementia, we employed a previously developed retinal image analysis method for cerebral white matter hyperintensities (WMH) using magnetic resonance imaging (MRI), a gold standard with an accuracy of more than 90% [37,38]. The WMH risk scores were produced using the Lau et al. (2019) [37] model and validated by Zee et al. (2021) [38]. For depression, we have also shown from our recent study on major depressive disorder patients that retinal image analysis can identify early depression cases in the community [39]. We have entered 310 psychiatrist-diagnosed major depressive disorder cases from a psychiatric hospital. Age- and gender-matched controls with low depression risk (confirmed via PHQ-9) were recruited from the community. Retinal images were captured using a non-mydriatic fundus camera. The classification model’s performance was evaluated using 10-fold cross-validation with a sensitivity of 98.1%, specificity of 99.3%, and an area under the ROC curve of 0.99, distinguishing MDD cases from controls.

## 3. Results

### 3.1. Primary Dataset and Validation Dataset

The primary dataset comprised 1480 colour fundus retinal images (Table 2). Among them, 453 (30.61%) images were labelled as referable AMD, and 1027 (69.39%) were labelled as control. Among images with referable AMD, 213 (14.39%) were labelled as neovascular AMD and 240 (16.22%) as non-neovascular AMD. The external validation dataset consisted of two sub-datasets, totalling 238 images: 41 with NVAMD, 48 with non-NVAMD, and 149 in the control group. The sub-dataset-1 comprised 168 colour fundus retinal images. Among them, 75 (44.64%) images were labelled as referable AMD, and 93 (55.36%) were labelled as control. Among images of referable AMD, 30 (17.86%) were labelled as neovascular AMD and 45 (26.78%) as non-neovascular AMD. Sub-dataset 2 consisted of 70 colour fundus retinal images. Among them, 11 (15.71%) images were labelled as neovascular AMD, 3 (4.29%) as non-neovascular AMD, and 56 (80.00%) as control.

### 3.2. Internal 10-Fold Cross-Validation

**Figure 2:**
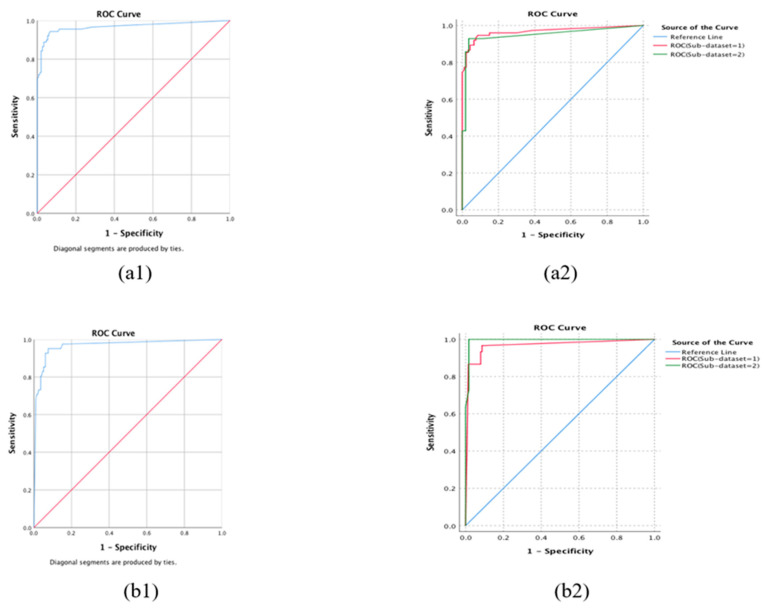
The performance of AMD detection in the validation dataset. (**a1**,**a2**) Referable AMD and (**b1**,**b2**) Neovascular AMD. 1: ROC curve in the validation dataset; 2: ROC curves in the validation sub-dataset-1 and sub-dataset-2.

The performance of ARIA in differentiating categories in 10-fold cross-validation is shown in Table 3 and Figure 2. The ARIA achieved sensitivities, specificities, and accuracies of 97.4%, 96.8%, and 97.0%, respectively, for the detection of referable AMD. To detect neovascular AMD, ARIA achieved a sensitivity of 98.1%, a specificity of 96.1%, and an accuracy of 96.4%.

### 3.3. External Validation

In the validation (Table 4 and Figure 2), our model maintained high performance in detecting referable AMD, with 85.4% sensitivity, 97.3% specificity, 92.9% accuracy, and an AUC of 0.967. Our algorithms achieved sensitivities of 85.3% and 85.7%, specificities of 96.8% and 98.2%, accuracies of 91.7% and 95.7%, and AUCs of 0.968 and 0.950 in sub-datasets 1 and 2, respectively. In comparison, there is no statistically significant difference in the performance of the two sub-datasets (*p* = 0.704).

Our model demonstrated strong capability to identify neovascular AMD, with 92.7% sensitivity, 93.9% specificity, 93.7% accuracy, and an AUC of 0.967. Our algorithms achieved sensitivities of 90.0% and 100.0%, specificities of 92.0% and 98.3%, accuracies of 91.7% and 98.6%, and AUCs of 0.967 and 0.996, respectively, in sub-datasets 1 and 2. Similarly, there is no statistically significant difference in the performance of the two sub-datasets (*p* = 0.213).

### 3.4. Segmentation—A Visual Presentation of an Explainable AI System

**Figure 3:**
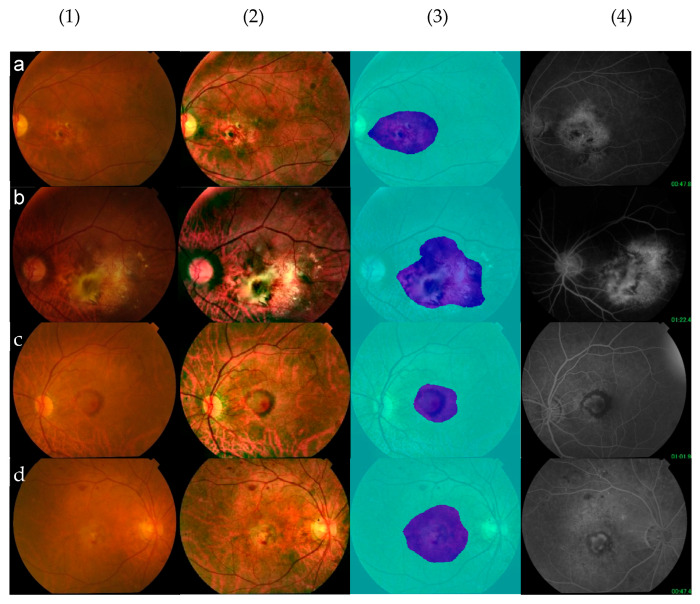
Examples of segmentation. (**a**–**d**) Eyes with neovascular AMD: (1) an original image; (2) images after pre-processing; (3) automatic segmentation; and (4) corresponding FA images (**a**–**d**).

To segment CNV, we used 213 images with neovascular AMD in the primary dataset to train our algorithms. Thirty images in the validation dataset were used for the external validation. Finally, we achieved global accuracy of 93.03%, mean accuracy of 91.83%, mean IoU of 68.7%, weighted IoU of 89.63%, and mean BFScore of 67.77% (Table 5). The examples of CNV segmentation are shown in Figure 3.

The visual presentation of the area of neovascular AMD is designed to provide users with greater confidence in their decision-making processes when using the AI-based retinal image analysis system in practice. It increases the interpretability and transparency of the decision by allowing the neovascularisation region to be visualised. It also helps us track the changes in neovascular AMD for longitudinal assessment of treatment outcomes.

### 3.5. Association with WMH as a Risk Factor for Dementia and Depression

We included 56 individuals with AMD (37 with neovascular AMD) and 195 controls in the analysis. The mean probabilities of having moderate-to-severe WMH in the control group, AMD, and neovascular AMD are 0.484 (95% CI, 0.474 to 0.494), 0.536 (95% CI, 0.525 to 0.547), and 0.559 (95% CI, 0.545 to 0.573), respectively. The control group has a significantly lower risk of WMH for both AMD (*p* < 0.001) and neovascular AMD (*p* < 0.001). The difference in WMH risk between AMD and neovascular AMD is not significant (*p* = 0.235). The risk of WMH in the control group is considered low, as the upper boundary of the 95% confidence interval is below 0.5. However, both AMD and neovascular AMD groups have significantly higher risks of WMH, with the lower boundary of the 95% confidence intervals exceeding 0.5.

For the depression risk, the mean probabilities of having a moderate or high risk of depression for the control groups, AMD, and neovascular AMD are 0.379 (95% CI, 0.365 to 0.394), 0.423 (95% CI, 0.400 to 0.446), and 0.442 (95% CI, 0.414 to 0.470), respectively. The control group has a significantly lower risk of depression than that of the AMD (*p* = 0.013) and neovascular AMD (*p* = 0.002). The difference with respect to the risk of depression between AMD and neovascular AMD is not significant (*p* = 0.655). However, there are significant differences in depression risk between the control group and the two AMD groups. It is noted that the estimated risk of depression is considered low, as the upper bound of the 95% confidence intervals for all three groups is less than 0.5.

## 4. Discussion

The study results demonstrate that our method has the potential to identify individuals with referable AMD for early intervention and those with neovascular AMD for urgent referral and prompt treatment, thereby preserving visual function. Our methods also demonstrated excellent classification performance on wide-field retinal images, indicating that they can be applied to images from diverse sources to facilitate translation in clinical settings. Furthermore, it has the potential to facilitate teleophthalmology or telemedicine in combination with colour fundus cameras, especially in rural communities where access to advanced ophthalmic devices such as FA and OCT is limited or medical care manpower is limited.

We automatically segment CNV on colour fundus retinal images using the reference standard of fundus angiography. To the best of our knowledge, this is the first study to automatically segment CNV by mapping the CNV segmentation from fundus angiography onto fundus retinal images to improve the accuracy of automatic segmentation. The algorithms demonstrated excellent performance, even compared with other studies that segmented CNV only on advanced ocular imaging, which offer more precise visualisation of CNV (Table 6). Tsai et al. [40] delineated and quantified CNVs based on fluorescence leakage characteristics using a random-walk algorithm on FA images. The results showed the average accuracy of CNV delineation was 83.26%. In another study, they developed two deep learning networks to detect PCV on FA images: an attention-gated convolutional neural network (AG-CNN) for detection and an attention-gated recurrent neural network (AG-PCVNet) for segmentation [41]. The AG-CNN model attained image-level and patient-level classification accuracies of 82.80% and 86.21%, respectively. The segmentation model, AG-PCVNet, yielded a balanced accuracy of 81.132% and a Dice score of 0.54. Separately, a fully automated algorithm by Wang et al., based on OCTA, demonstrated high efficacy in diagnosing CNV, achieving 100% sensitivity and 95% specificity in distinguishing CNV cases from controls [42]. The model achieved a mean Intersection over Union (mIoU) of 0.88 for the segmentation of the CNV membrane. Compared to the performance of their studies, our algorithm achieved high global and mean accuracy (>90%). It outperformed studies using FA images, despite the use of colour fundus retinal images, which posed greater challenges. Since fundus photography is quick, simple, and cost-effective, our approach can serve as a screening tool to help directly assess the potential severity of neovascular AMD and the urgency of referral in real time, and to assist clinicians in focusing on the corresponding field during operative and subsequent OCT and FA examinations. Moreover, changes in CNV size after anti-vascular endothelial growth factor (anti-VEGF) treatment correlated with changes in BCVA [43]. Thus, for prognostic evaluations, prompt assessment of CNV status will be crucial to preserving vision. Our study suggested that this method may be a quick and accessible tool for non-invasively monitoring CNV over time, enabling early detection of lesion changes and tracking the response to anti-VEGF agents.

Our results are comparable to those of other studies in distinguishing between normal/early and intermediate/late AMD. Agurto et al. (2011) achieved a sensitivity of 0.94, a specificity of 0.50, and an AUC of 0.84 [44]. Burlina et al. (2017) employed a deep convolutional neural network, achieving accuracies of 88.4% to 91.6% and AUCs of 0.94 to 0.96 [10]. Ting et al. (2017) achieved a sensitivity of 93.2%, a specificity of 88.7%, and an AUC of 0.931 [45]. The performance of a self-supervised network was found to be 87% in the study by Yellapragada et al. (2022), demonstrating slightly worse than supervised-trained (90%), published supervised network (92%), and an ophthalmologist (96%) [46]. Our overall performance on the validation dataset is slightly better than theirs (accuracy = 92.9%, AUC = 0.967), indicating that our algorithm can effectively classify unseen images from other sources. Similar to our study, which detected referable AMD and neovascular AMD, Heo et al. (2020) [47] conducted a three-class classification (control vs. dry AMD vs. neovascular AMD) using the Visual Geometry Group with 16 layers (VGG16) model. They achieved 90.86% accuracy with pre-processing in five-fold cross-validation [47]. Our study also showed comparable results even in the external validation.

We have demonstrated that referable AMD has a significantly higher risk of WMH (a significant risk factor for dementia) and depression. This result aligns with other clinical studies. However, we demonstrated that AI-based retinal image analysis enables risk assessment across multiple indications, including AMD and its associated brain health risks, using a single set of retinal images. Future studies can be conducted to evaluate if early detection and intervention of AMD may have a significant impact on both vision and brain health conditions, as measured by risks of WMH and depression outcomes.

Our study has several advantages over other studies. (1) We considered not only referable AMD but also neovascular AMD, which could indicate the severity of AMD for further referral and management. (2) The automatic segmentation of CNV can also facilitate further ocular examinations and treatment promptly. (3) Our reference standard is based on retinal images, FA, and clinical diagnosis, which may be more accurate than the manual assessment based only on retinal images. (4) Our method is a combination of a transfer net, InceptionResNetV2 convolutional neural network, and AIRA algorithm, which is more comprehensive. We extracted associated features from retinal images, going beyond the limitations of using only a deep learning network, a black box which is difficult to interpret [48]. (5) We applied 10-fold cross-validation and two external validation datasets comprising images from various cameras to assess our models and the generalisation and practicality of our algorithms across different settings. (6) We demonstrated that referable AMD and neovascular AMD are significantly worse than controls with respect to risk of dementia and depression. It highlights the importance of implementing effective interventions for AMD as early as possible.

However, limitations of this study include that our method showed moderate sensitivity (above 85%) in detecting referable AMD during external validation. This is expected, as non-mydriatic and wide-field images were included in the validation dataset, which presented significant challenges in detecting subtle drusen. The Mean IoU (68.7%) and Mean BFScore (67.77%) also showed moderate precision in delineating CNV boundaries. Moreover, our validation dataset has a relatively small number of images. It is limited to Chinese populations, whereas the patients at Aier Eye Hospital come from northern areas, unlike the eastern areas of the training dataset in China. Also, the validation dataset used wide-field retinal images and different camera types for further application in different scenarios. In future work, increasing the sample sizes of non-mydriatic and wide-field images and including patients from various ethnicities could further refine and improve the performance of our methods.

## 5. Conclusions

In conclusion, we presented an AI-based retinal image analysis method for detecting AMD, which aligns closely with clinical decision-making processes and serves as an efficient, cost-effective tool from a clinical practice perspective. Additionally, it provides crucial information on CNV, helping clinicians assess AMD severity, particularly in settings where OCT and FA are not readily available. Ultimately, our approach addresses the three-stage AMD detection process: screening for referable AMD, detecting neovascular AMD for urgent referral, and segmenting CNV as an outcome of an explainable AI system to supplement interpretation and assist with further diagnosis, ocular examinations, and treatments. Since there are effective treatments for AMD, future studies may reveal potential benefits for AMD screening that extend beyond vision improvement and also enhance brain health outcomes.

## Figures and Tables

**Table 1:** Definition of different stages of AMD.

Classification	Category	Stage	Definition
Control	1	No AMD	No drusen or only small drusen 63 mm, and no pigment abnormalities
	2	Early AMD	Medium drusen > 63 mm and ≤125 mm, and no pigment abnormalities
Referable AMD	3	Intermediate AMD	Large drusen > 125 mm or any pigment abnormalities
	4	Advanced AMD	Neovascular AMD or geographical atrophy

**Table 2:** The summary of images in the primary dataset and the validation datasets.

	Category	Primary Dataset	Validation Datasets		
			Sub-Dataset 1 *	Sub-Dataset 2 *	Total
Referable AMD		453 (30.61)	75 (44.64)	14 (20.00)	89 (37.39)
	NVAMD	213 (14.39)	30 (17.86)	11(15.71)	41 (17.23)
	Non-NVAMD	240 (16.22)	45 (26.78)	3 (4.29)	48 (20.16)
Control		1027 (69.39)	93 (55.36)	56 (80.00)	149 (62.61)
Total		1480 (100)	168(100)	70 (100)	238 (100)

* Sub-dataset 1 from Zhongshan Hospital; Sub-dataset 2 from Shenzhen Aier Eye Hospital. NVAMD: Neovascular AMD; Non-NVAMD: Non-neovascular AMD.

**Table 3:** Results for the 10-fold cross-validation of the primary classification model.

	Se (%)	Sp (%)	Acc (%)
Referable AMD vs. Control	97.4	96.8	97.0
NVAMD vs. (Non-NVAMD + Control)	98.1	96.1	96.4

NVAMD: neovascular AMD; Non-NVAMD: Non-neovascular AMD; Se: Sensitivity; Sp: Specificity; Acc: Accuracy.

**Table 4:** Results of the validation of Referable AMD and Neovascular AMD.

	Confusion Matrix			Se	Sp	Acc	AUC	*p*
Referable AMO vs. Control		1	0					
	1	76	4	85.4	97.3	92.9	0.967	-
	0	13	145					
Subgroups		1	0					0.704
Sub-dataset 1	1	64	3	85.3	96.8	91.7	0.968	
	0	11	90					
Sub-dataset 2	1	12	1	85.7	98.2	95.7	0.950	
	0	2	55					
NVAMD vs. (Non-NVA + Control)		1	0					
	1	38	12	92.7	93.9	93.7	0.967	-
	0	3	185					
Subgroups		1	0					0.213
Sub-dataset 1	1	27	11	90.0	92.0	91.7	0.967	
	0	3	127					
Sub-dataset 2	1	11	1	100.0	98.3	98.6	0.996	
	0	0	58					

NVAMD: neovascular AMD; Non-NVAMD: Non-neovascular AMD; Se: Sensitivity; Sp: Specificity; Acc: Accuracy. Confusion matrix: row = predicted condition; column = actual condition.

**Table 5:** The performance of automatic segmentation of CNV in the images with neovascular AMD.

	Global Acc	Mean Acc	Mean IoU	Weighted IoU	Mean BF Score
Segmentation	93.03%	91.83%	68.7%	89.63%	67.77%

CNV: Choroidal neovascularisation; Acc: Accuracy; IoU: intersection over union; BF: boundary FI.

**Table 6:** The comparison of studies with various methods to delineate CNV and detect referable AMD and neovascular AMD.

	Data Source	Method	Performance
CNV Delineation			
Tsai et al. [40]	Fluorescein angiography images	Random walk algorithm	Accuracy = 83.26%
Wang et al. [42]	Projection-resolved optical coherence tomographic angiography (PR-OCTA)	Convolutional neural networks (CNNs)	Mean intersection over the union = 0.88
Our study	Colour fundus retinal images		Global accuracy = 93.03%; Weighted IoU = 89.63%
Referable AMD Detection			
Agurto et al. [44]	Retinal digital photographs	A computer-aided algorithm	Sensitivity = 0.94; Specificity = 0.50; AUC = 0.84
Burlina et al. [10].	Fundus images	Deep convolutional neural networks	Accuracy: 88.4% to 91.6%; AUC: 0.94 to 0.96
Ting et al. [45]	Retinal images	Deep learning system (DLS)	Sensitivity = 93.2%; Specificity = 88.7%; AUC = 0.931
Yellapragada et al. [46]	Fundus photographs	Deep neural network with self-supervised Non-Parametric Instance Discrimination (NPID)	Self-supervised-trained network: Accuracy = 87%; Supervised-trained network: Accuracy = 90%
Our study	Colour fundus retinal images	AI-based Retinal Image Analysis (ARIA)	Sensitivity = 97.4%; Specificity = 96.8%; Accuracy = 97.0%
Neovascular AMD Detection			
Heo et al. [47]	Fundus photographs	Visual Geometry Group with 16 layers (VGG16) model of convolutional neural networks	Normal vs. nAMD: accuracy = 0.9099; dAMD vs. nAMD: accuracy = 0.7601
Our study	Colour fundus retinal images	AI-based Retinal Image Analysis (ARIA)	nAMD vs. (Non-nAMD+Normal): Sensitivity = 98.1%; Specificity = 96.1%; Accuracy = 96.4%

AUC: area under the curve; nAMD: neovascular age-related macular degeneration; dAMD: dry age-related macular degeneration.

## Data Availability

The original contributions presented in this study are included in the article. Further inquiries can be directed to the first author.
